# Quality of life after antegrade intramedullary nail fixation of humeral fractures: a survey in a selected cohort of Brazilian patients

**DOI:** 10.1186/s13037-018-0150-8

**Published:** 2018-03-12

**Authors:** Luiz Fernando Cocco, Benno Ejnisman, Paulo Santoro Belangero, Moises Cohen, Fernando Baldy dos Reis

**Affiliations:** Department of Orthopedics and Traumatology, (DOT/UNIFESP)-Escola Paulista de Medicina, Universidade Federal de São Paulo, Rua Borges Lagoa, 778, São Paulo, SP CEP 04024-002 Brazil

**Keywords:** WORC, Shoulder function, Rotator cuff, Humeral intramedullary nailing

## Abstract

**Background:**

The treatment of humeral fractures remains controversial. Systematic reviews demonstrate similar results between dynamic compression plating and locked intramedullary nailing in the surgical treatment of these fractures. However, it appears that antegrade intramedullary nailing causes higher residual pain in the shoulder. The proposal of this work is to evaluate through the WORC protocol (Western Ontario Rotator Cuff Index) the consequences in the quality of life of patients submitted to osteosynthesis of the humerus with antegrade locked intramedullary nailing.

**Methods:**

This work is a cohort retrospective study in addition to the application of a questionnaire for self-rated quality of life with its 05 domains (WORC - Western Ontario Rotator Cuff Index) for patients (*N* = 26) classified in the Trauma Sector of the Department of Orthopedics and Traumatology of the Federal University of São Paulo (DOT/UNIFESP) submitted to Humerus Osteosynthesis with Antegrade Locked Intramedullary Nailing. There was also the inclusion of data related to the time since surgery, age, sex, surgical laterality, dominance among members and work leave, which were not considered in the original protocol. After, the data were statistically assessed to evaluate the association between numerical and categorical variables.

**Results:**

The overall WORC score was 82.75 ± 17.00 (Mean ± SD) and was not different considering sex, age and postoperative period. Among the WORC domains, both Work and Sport / Recreation Protocols were the most unfavorable factors in the evaluation of patients. Although not statistically significant, those who had the procedure on the dominant side presented a lower quality of life score than those who had the surgery on the non-dominant side. Although non-significant again, those who were away from work had an overall lower quality of life score than those who were not.

**Conclusions:**

The WORC Quality of Life Protocol shows good results for evaluating patients submitted to humerus osteosynthesis with antegrade locked intramedullary nailing. The data stratified by domains were good, however, Work and Sport/Recreation domains showed the lowest means compared to the other domains.

**Trial registration:**

Research Ethics Commitee (CEP 0676/2016) and Plataforma Brasil 56381216.3.0000.550. CAAE: 56381216.3.0000.5505.

## Background

Humerus fractures are frequent, corresponding to approximately 3% of all fractures in adults. The treatment for fractures of the proximal and diaphyseal regions remains controversial, and there is no evidence for the best treatment and whether the surgical procedures are superior to non-surgical ones [1–3]. Therefore, periodic evaluations and follow-up of patients submitted to any type of treatment to correct these fractures are fundamental. The follow-up can be performed through patient care, and through the improvements and the development of new techniques already described in the literature.

Among the choices for surgical treatment, the most commonly used implants are the dynamic compression plating and locked intramedullary nailing [4]. Again, most studies did not demonstrate significant differences between rates of consolidation of humeral fractures using any of these two different implants [5, 6]. However, humerus antegrade access nailing was related to greater complaints of residual pain, movement limitation and subacromial impact on the shoulder [5]. The causes were related to the incision of the rotator cuff for implant fixation. On the other hand, results analyzed by Kurup et al. [5] demonstrated no differences for the patients’ functional capacity of the operated limb after six months of treatment using the American Shoulder and Elbow Surgeons (ASES) index, regardless of the implant used. According to Schmidutz et al. [7], the evaluation and interpretation of shoulder and upper limb injuries is controversial and subjective, and should be considered beyond the clinical examination. Subjective variables such as emotional aspects, sports, recreation or motivational habits of each patient should be interpreted differently.

Several scores protocols on quality of life have already been proposed to consider these differences: SPADI (Shoulder Pain And Disability Index), DASH (Disabilities of the Arm, Shoulder and Hand Questionnaire), ASES (American Shoulder and Elbow Surgeons), SRQ (Self Reporting Questionnaire), WOOS (Western Ontario Osteoarthritis of the Shoulder), SSRS (Subjective Shoulder Rating System), CMS (Constant Murley Score), MSQ (Munich Shoulder Questionnaire), among others [6]. In addition, indirect gains or withdrawals related to labor issues might interfere with the self-assessment protocols of some patients [8]. The WORC protocol, with its 5 domains (physical symptoms, sports/recreation, work, lifestyle, emotions) facilitates the comprehension of these aspects for evaluating the results of the quality of life of patients submitted to an incision in the rotator cuff for intramedullary nail fixation.

Therefore, the objective of this work was to do a follow up in patients that were submitted to surgeries through humeral fractures fixation using locked antegrade intramedullary nailing for a minimum of 6 months, using the WORC Quality of Life Protocol to gather data aiming for a better understanding of patients’ well being after the surgery.

## Methods

A retrospective review of patients cataloged in the DOT / UNIFESP, Department of Orthopedics and Traumatology of the Federal University of São Paulo (Brazil) submitted to Humerus Osteosynthesis using Locked Intramedullary Nailing with antegrade access was performed.

### Inclusion criteria

Adults (older than 18 years), both male and female, with a fracture in the diaphyseal region or immediately below the humeral head, with simple or complex features, but not extending to the shoulder or elbow joints, underwent osteosynthesis of the humerus with an antegrade locked intramedullary nailing, for a minimum of 06 months earlier and have not undergone other forms of treatment (surgical or otherwise) prior to arrival at the Hospital. They did not report diseases or other shoulder surgeries prior to receiving the implant. They agreed to the terms of the Free and Informed Consent Form (FICF) as recorded in the University Ethics Committee.

### Exclusion criteria

These were based on suspected clinical and radiographic pathological fractures such as metastatic fractures or bone infection of the humerus, and signs of neurological or vascular lesions in the initial fracture diagnosis, such as radial neuropraxia, brachial plexus injuries, changes in the wrist or distal perfusion to the fracture, fractures in other body parts, fractures of the proximal humerus with more than 2 parts by the classification of Neer CS, 1970 [8], or associated abdominal, thoracic or intracranial lesions. Because it is a protocol of self-assessment of quality of life, additional injuries not related to the humerus fracture could influence the participant’s response to the questionnaire which was not related to the evaluation of the rotator cuff, affecting the study objective. In addition, were also excluded those who did not agree with the conditions of the FICF.

The WORC questionnaire consists of 21 items contained in five different domains (physical symptoms, sports/recreation, work, lifestyle and emotions). All items have the same weight value and each one can be scored from 0 to 100 mm according to a visual scale. The total questionnaire can range from 0 to 2100. To facilitate the interpretation of the final score, the authors of the original version recommended that the data should be converted to the percentage. A score of 0 is the worst possible outcome and 100% implies no reduction in the quality of life. The questionnaire was validated to Portuguese language and demonstrates equivalence to DASH, UCLA, Constant Score, Short-WORC and SF-36 Physical domains [8–13].

Although not included in the original WORC protocol proposed by Kirkley et al. [9], we included baseline information and surgery-related data such as age, gender, time since last surgery, surgical laterality, dominance among the limbs, and whether the patients were on leave at the time of filling out the questionnaire. This information was obtained in the same interview and arranged as an appendix to the original questionnaire, not influencing the participants’ input or interpretation of the original questions. Additional results were compared with WORC score when statistical analysis was performed.

The data were evaluated in two ways: individualized by domain and as a general score by the patient according to the WORC questionnaire. The information included in the identification and registration form was interpreted separately. Subsequently, they were compared with general scores obtained in the WORC questionnaire.

We used the Mann-Whitney test to evaluate the association between numerical variables (age, time since last surgery and quality of life scores) and the Chi-Square and Fisher Tests for the categorical variables (sex, laterality, dominance, work leave). We used *p* < 0.05 for significance. The results of the five domains and general WORC scores are presented through simple means and standard deviations and then sorted according to prevalence.

**Fig. 1:**
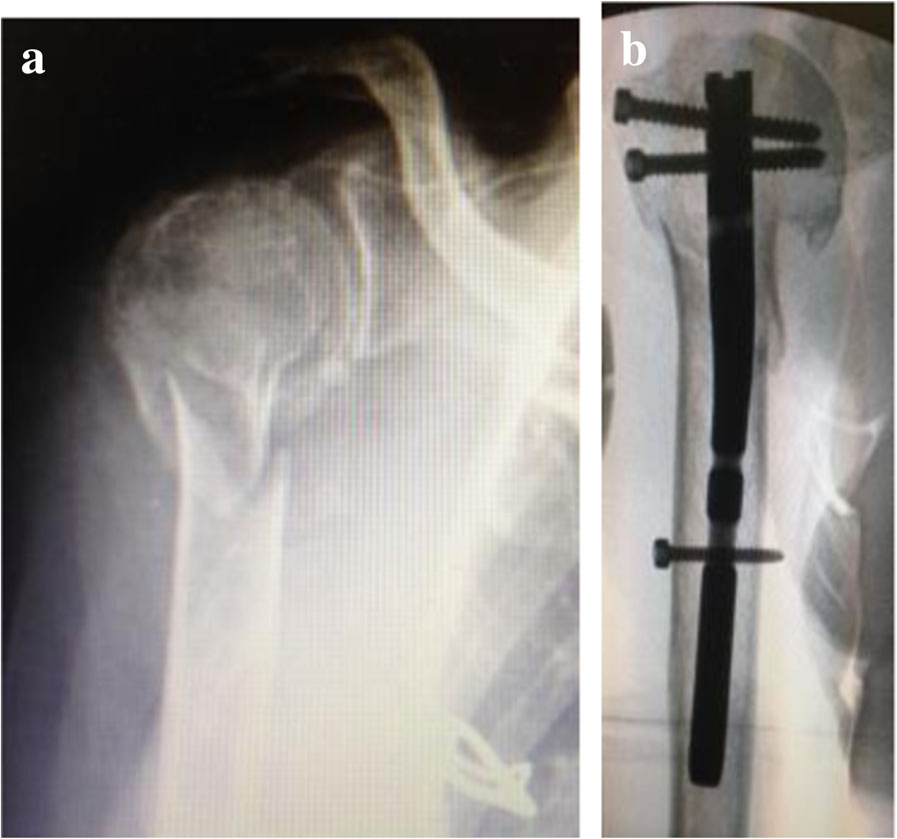
Radiographies of the right shoulder (**a**) of a 68-year patient with fracture in two parts of the humerus proximal region (Neer classification) and treated with short-locked intramedulary nailing (**b**), with implant placement through the rotator cuff

**Fig. 2:**
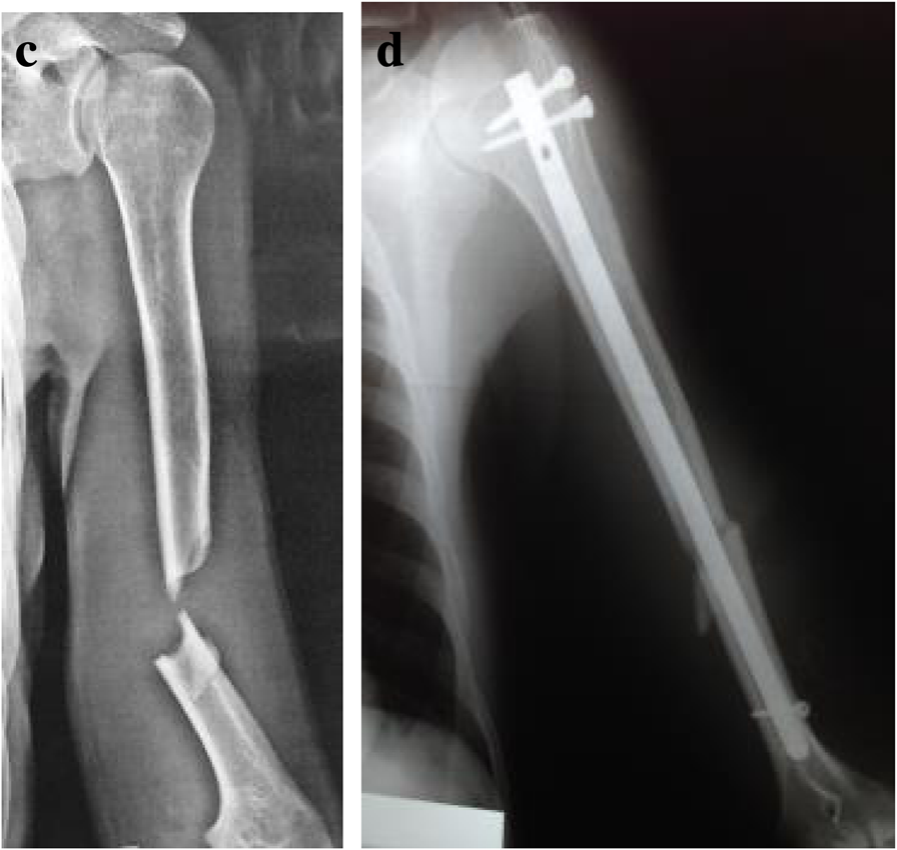
Radiographies of the left arm (**c**) of a 40 year-old patient with diaphyseal humerus fracture treated with long-locked intramedulary nailing (**d**), with implant placement through the rotator cuff

The patients were operated by the same group of surgeons, belonging to the Trauma Sector at DOT/UNIFESP – Brazil. It was used blocked intramedullary nails developed for entry at the humerus apex, after opening through the long axis of the fibers of the rotator cuff towards the abdominal muscle, avoiding the insertion of the tendons in the larger tubercle. The choice of stem length was based on the anatomical location of the fracture at the initial x-ray examination. Short stems were indicated for fractures fixations affecting the proximal humerus region up to approximately the uppermost insertion of the pectoralis major muscle, or those classified by Neer in up to 02 parts. The long stem was indicated for fractures fixation below the inferior border of the pectoralis major muscle, up to the region of the humeral supracondylar ridges, with a sufficiently safe distance for placement of at least 01 of the distal implant blocks (Figs. 1 and 2). The rotator cuff closure is made with unabsorbable surgery strings, through simple points that re-approximate the extruded fibers, completely covering the humeral head.

The rehabilitation was followed by physiotherapists belonging to the Trauma Sector at DOT/UNIFESP, basically orienting the patient to remain with a simple sling on the operated side for a good analgesic condition. Next, the patient was encouraged to stimulate the shoulder arch and the elbow as soon as possible according to pain sensitivity. Activities of strengthening and elongation of external rotators and scapular stabilizers were initiated after pain control.

## Results

### Sample characteristics

Thirty (30) patients were contacted by telephone and 26 of them attended the interview, responding to the questionnaire only once.

Gender: 16 men and 10 women.

Age: 20 to 89 years (49.8 ± 20.1 years).

Dominance: 24 right-sided and 2 left-sided.

Last Surgery: 06 months up to 06 years (mean of 3.0 ± 1.8 years).

Laterality: 16 right sided and 10 left sided.

Surgical Dominance and Laterality: 12 patients with surgery on the dominant side and 14 on the non-dominant side.

Work leave at the time of the questionnaire: 5 patients.

Twenty-one (21) patients had diaphyseal fractures and 5 patients had the surgical neck treated with antegrade locked intramedullary nailing.

The overall score varied from 41.26 to 99.83 (mean 82.75 ± 17.00), and was not different considering sex, age and postoperative period (Table 1). Among the domains that make up the Protocol for the Evaluation of Quality of Life (WORC), Work and Sports/Recreation were the most unfavorable factors, with a mean of 10.54 ± 9.00 and 7.36 ± 8.44, respectively (Table 1). These were followed by Physical Symptoms (mean of 6.84 ± 6.97), Lifestyle (mean of 6.36 ± 8.54) and Emotions (mean of 5.14 ± 7.21) (Table 1).

**Table 1:** Results of the Quality of Life Protocol (WORC) and its 5 domains. Means ± SD are shown (*N* = 26)

Patients	Physical Symptoms	Sports/ Recreation	Work	Life Style	Emotion	Summary	Summary %
1	11,55	18,2	15,7	8,35	5,9	59,7	71,50%
2	5,7	12,2	11,25	10,3	2,35	41,8	80,09%
3	0,35	1,05	0	0	0	1,4	99,30%
4	0	1,35	3,1	1,7	0	6,15	97,07%
5	16,35	17,75	14,5	4,15	6,55	59,3	71,76%
6	2,7	11,7	8,15	4,5	7,5	34,55	83,54%
7	0	0,35	0,7	0	0	1,05	99,50%
8	4,1	6,55	20,4	15,9	2,4	49,35	76,50%
9	0	1,35	0,35	0,35	0	2,05	99,02%
10	0	1	4,5	0	0	5,5	97,38%
11	16,75	28,5	24,2	17,75	13,5	100,7	52,04%
12	17,45	10,65	14,15	2,4	3,8	48,45	76,92%
13	11,95	3,05	10,25	2,45	8,25	35,95	82,88%
14	4,1	3,4	13,7	0	0	21,2	89,90%
15	24,15	23,8	27	27,2	21,2	123,35	41,26%
16	17,75	1,4	28,55	28,25	28,5	104,45	50,26%
17	1,05	0	0,7	0,35	1,05	3,15	98,50%
18	0	0	0	0,35	0	0,35	99,83%
19	4,45	1,05	16,8	7,6	3,7	33,6	84%
20	6,5	22,8	9,6	0,7	8,9	48,5	76,90%
21	3,1	0	0,35	0	1,05	4,5	97,85%
22	0,35	0	0	0	0,35	0,7	99,66%
23	4,7	6,15	7,45	4,05	3,1	25,45	87,88%
24	4,45	4,75	8,2	0,7	0	18,1	91,38%
25	8,2	4,8	10,05	8,35	1,05	32,45	84,54%
26	12,15	9,6	24,4	19,95	14,4	80,5	61,60%
Total	177,85	191,45	274,05	165,35	133,55	942,25	82,75%
Total %	18,87%	20,31%	29,08%	17,54%	14,17%	100%	
Mean	6,84	7,36	10,54	6,36	5,14	36,24	83%
Standard Deviation	6,97	8,44	9,00	8,54	7,21	35,08	17%

Although non-significant, those who underwent the surgery on the dominant side (patients 1, 2, 4, 6, 7, 9, 15, 16, 20, 24, 25 and 26) presented a quality of life score lower (78.1 ± 18.9) than those who had surgery on the non-dominant side (86.7 ± 13.9; *p* = 0.20). Again, although non-significant, those who were away from work (patients 1, 5, 15, 18 and 26) presented a general lower quality of life score (69.2 ± 21.1) than those who were not (85.9 ± 14.2, *p* = 0.10).

## Discussion

We used a quality of life (WORC) protocol, to evaluate patients undergoing humerus osteosynthesis with antegrade intramedullary nailing linked to the rotator cuff function. We analyzed the domains average that is part of this protocol, observing which of them could be considered critical for the indication of this type of surgery, besides factors related to surgical laterality and dominance among members, gender, age, and work leave.

The rotator cuff violation for antegrade intramedullary nailing procedure is a mandatory surgical procedure, which can be considered a risk factor for residual shoulder functions. In the sample studied here, we used only implants with access to the center of the humeral head, reserving the area of tendinous insertion of the rotator cuff over the larger tubercle. The protocol analysis was satisfactory about the consequences of surgical manipulation and residual shoulder function in the operated patients. The overall final results of the protocol suggest that quality of life is not significantly impaired after performing the surgery.

In addition, the individualized analysis by domains that compose the WORC questionnaire did not demonstrate discrepant values and can be considered satisfactory within its particularities. However, Work and Sports/Recreation were the domains with the lowest means and, in this sample, can be considered as critical factors for indication of intramedullary nails with antegrade surgical access for certain patients, such as sportsmen or workers with great demand in the upper parts of the body.

The information included in the original protocol proposed by Kirkley et al. [9] about surgical laterality and dominance among members, sex, age, time since last surgery and work leave were not statistically significant in the results reported here. However, surgery on the dominant limb and work leave showed a tendency to worsen the outcomes in the quality of life. Any conflicts of interest from the participants during the self-assessment may explain the higher weight of the work domain, which was presenting the greatest impact factor on the final mean of the WORC protocol. In the same way the functional restriction, even temporary, of the dominant member in the daily life can be more impacting when compared to the non-dominant member.

Although the use of intramedullary antegrade nailing for the treatment of humerus fractures is not performed with the same frequency as seen for tibia or femur, the number of participants here restricts the results to the population studied in this work. Moreover, the results did not present enough statistical power to conclude the hypotheses tested. Multicentric studies are a good way to select a larger number of patients to study this treatment. In addition, the patients answered the questionnaire only once, and at least after 06 months of postoperative evolution. Functional evaluations of the shoulder can change with time, directly affecting the values measured at the time of data collection. Yet, the data obtained in this analysis and compared to other studies [14–16], reinforce the use of antegrade locked intramedullary nailing for the treatment of humerus fractures, with no significant impact on the quality of life assessed by the WORC protocol.

## Conclusions

The WORC Quality of Life Protocol shows good results for evaluating patients submitted to humerus osteosynthesis with antegrade locked intramedullary nailing, with an overall mean of 82.7 after the treatment. The data stratified by domains were also good, with high averages within each profile evaluated. Work and Sport/Recreation, although with a good average score, were considered areas of high risk for indication of intramedullary nailing for certain patients.

## Abbreviations

ASES: American Shoulder and Elbow Surgeons
CMS: Constant Murley Score
DASH: Disabilities of the Arm, Shoulder and Hand Questionnaire
DOT: Department of Traumatology
FICF: Free and Informed Consent Form
MSQ: Munich Shoulder Questionnaire
SPADI: Shoulder Pain and Disability Index
SRQ: Self Reporting Questionnaire
SSRS: Subjective Shoulder Rating System
WOOS: Western Ontario Osteoarthritis of the Shoulder
WORC: Western Ontario Rotator Cuff Index
